# Skeletal Metastases of Unknown Primary: Biological Landscape and Clinical Overview

**DOI:** 10.3390/cancers11091270

**Published:** 2019-08-29

**Authors:** Antonella Argentiero, Antonio Giovanni Solimando, Oronzo Brunetti, Angela Calabrese, Francesco Pantano, Michele Iuliani, Daniele Santini, Nicola Silvestris, Angelo Vacca

**Affiliations:** 1 Medical Oncology Unit, IRCCS Istituto Tumori “Giovanni Paolo II” of Bari, Viale Orazio Flacco, 65, 70124 Bari, Italy; 2 Department of Biomedical Sciences and Human Oncology, University of Bari “Ando Moro”, Piazza Giulio Cesare, 11, 70124 Bari, Italy; 3 Department of Radiology, IRCCS Istituto Tumori “Giovanni Paolo II”, Viale Orazio Flacco, 65, 70124 Bari, Italy; 4 Medical Oncology, Campus Bio-Medico University of Rome, Álvaro del Portillo, 21, 00128 Rome, Italy

**Keywords:** skeletal metastases of unknown primary, SMUP, bone metastases, unknown primary tumor, bisphosphonates, bone markers, tumor microenvironment

## Abstract

Skeletal metastases of unknown primary (SMUP) represent a clinical challenge in dealing with patients diagnosed with bone metastases. Management of these patients has improved significantly in the past few years. however, it is fraught with a lack of evidence. While some patients have achieved impressive gains, a more systematic and tailored treatment is required. Nevertheless, in real-life practice, the outlook at the beginning of treatment for SMUP is decidedly somber. An incomplete translational relevance of pathological and clinical data on the mortality and morbidity rate has had unsatisfactory consequences for SMUP patients and their physicians. We examined several approaches to confront the available evidence; three key points emerged. The characterization of the SMUP biological profile is essential to driving clinical decisions by integrating genetic and molecular profiles into a multi-step diagnostic work-up. Nonetheless, a pragmatic investigation plan and therapy of SMUP cannot follow a single template; it must be adapted to different pathophysiological dynamics and coordinated with efforts of a systematic algorithm and high-quality data derived from statistically powered clinical trials. The discussion in this review points out that greater efforts are required to face the unmet needs present in SMUP patients in oncology.

## 1. Introduction

Skeletal metastases of unknown primary (SMUP) represent enigmatic rare metastatic tumor entities without anatomic primary sites identified. Cancer of unknown primary (CUP) accounts for 2% of all cancers characterized by an aggressive clinical outcome and poor response to chemotherapy [1,2]. Although almost all tumors can metastasize to the skeleton during their natural history, epithelial cancers are characterized by a particular propensity for this type of dissemination. However, modern diagnostic methods, including molecular investigations are not always sufficient to identify the primary site of the neoplasm in order to guide a targeted treatment. Multiple body regions involved are identified in more than 50% of individuals with CUP [3]. The skeleton is the third most common site of metastatic cancer after lung and liver, often representing the onset of unknown primary tumors in 8–23% of patients with poor prognostic clinical features [4,5,6,7,8]. Lung cancer is the most frequently identified primary tumor (25–67%) across all literature data. The other most frequent primary malignancies are multiple myeloma, prostate cancer, lymphoma, kidney, gastrointestinal tumor, and breast cancer. Nonetheless, the primary site of bone metastases often remains unidentified despite diagnostic investigations and autoptic examination (Table 1).

Literature data reported that the spine is the most common site of SMUP, followed by the pelvis and bones of the extremities [5]. Takagi et al. reported a large retrospective analysis of 286 SMUP and showed that 2/3 had multiple bone lesions without differences in localization, while a solitary bone metastases occurred in 32.5% [9]. Twenty-seven percent of patients presented three or more areas of localization. The number of bone metastatic sites is related to the prognosis of patients, with 39 months for solitary bone metastasis and 16 months for multiple sites (7 months for three or more areas). The primitive site following the diagnostic investigations was diagnosed in almost 89% of patients. In the residual 11% of the cases, the primary site remained unknown. The median overall-survival (mOS) of confirmed SMUP was 11 months compared to 20 months of the overall population, highlighting that bone metastases from unknown tumor represents a poor prognostic feature [9].

Interestingly, Hemminki et al. correlated a site-specific survival from 9,306 CUP patients with the metastatic site. The study highlighted shorter median OS of 203 patients with bone metastases (3 months) [8]. Clinical evidence is seldom available, and the data presented are often obtained from case series or retrospective studies with insufficient homogeneity. Nonetheless, a comprehensive overview of published data is summarized in Table 1. Additional unfavorable prognostic factors for CUP include male gender and an adenocarcinoma histotype.

## 2. Cancer Cell Homing to the Bone Marrow: Bridging the Gap Between the Malignancy and the Neighborhood

### 2.1. The Biological Landscape of Cancer Cells of Unknown Primary

The biological mechanisms underlying the tumors of unknown origin are still poorly understood. The identification of common etiological factors for a heterogeneous group of neoplasia, including different histotypes, represents a clinical challenge [19]. There are two main theories used to explain the biology of CUP. Remarkably, most of the primary cancers in the autoptic series measured less than 1 cm. The burned-out theory hypothesized that the cancer cells involute irrespective from the metastasis development due to a complex interplay between the tumor microenvironment and the molecular cancer features. Alternatively, the possibility of the existence of a peculiar progenitor cell, or rest cell, that might be the cell of origin of the metastatic site has been proposed [20]. These rest cells could undergo an incomplete migration to the designated tissue. Moreover, some histotypes such as germ cells, gonadal, thymic and lymphomas can physiologically arise anywhere in the various body compartments. Next, the elucidation of spatial and clonal heterogeneity shed more light on the genomic landscape. These genetic lesions can determine specific gene expression patterns. Intriguingly, tumor-initiating cells have the potential to trans-differentiate into multiple phenotypes [21,22,23,24]. To date, however, there are few studies and limited cases reported. The observation of an increased incidence of metastatic primitive adenocarcinoma of unknown origin in homozygous twins affected by primary immunodeficiency linked to the X chromosome led to the postulation of the presence of genetic abnormalities characteristic of CUP and their role in the process of metastasis [25,26]. The complete or partial loss of the short arm of chromosome 1, for example, has been found in several cases of neoplasia of unknown origin and seems to correlate with a particular capacity of the tumor to metastasize at a very early stage of its natural history, when it is not clinically detectable. A study published in 2010 based on the analysis of the Swedish Family Cancer Database supports that CUP may have a genetic basis [27]. The analysis showed that 2.8% of occult primary cases were familial (i.e., a parent and offspring were both diagnosed with occult primary cancer). In addition, CUP was associated with the occurrence of kidney and colorectal cancers in families, suggesting that these types of tumors are often the primary sites of the disease [27]. The incidence and the clinical relevance of the presence of overexpression of p53, b-cell lymphoma 2 (bcl-2), avian myelocytomatosis virus oncogene cellular homolog (c-myc), rat sarcoma viral oncogene homolog (ras), and human epidermal growth factor receptor 2 (HER-2) in neoplasms of an occult primitive site have not been assessed and the data reported in the literature are not complete [28,29,30,31,32].

### 2.2. Bone Dissemination Mechanisms: From Cell Biology to Metastatic Niche Physiopathology

Different approaches have been proposed in order to resolve the biologic complexity underlying the pathophysiologic step to bone dissemination. The first of the steps in the process of cancer spreading to the bone is the homing to the marrow microenvironment throughout the bloodstream of the tumor cells, via the neo-angiogenesis process, trough permissive bone marrow endothelial cells [33,34]. Remarkably, a prone microenvironment is involved in the cancer cycle, educating and hijacking the tumor niche that is to be colonized throughout a neoplastic permissive environment [35,36]. Ancillary to the cancer intrinsic mechanisms, peri-neoplastic infiltrates actively prime drug-resistance mechanisms both in solid and hematologic neoplasms expressing an osteotropic phenotype [37,38]. Therefore, a number of molecular actors have been considered as elements that drive the neoplastic cells to the bone environment, including karyotypic non-random abnormalities (i.e., t(11:22), t(15;19) [39], t12p, t(X;18), and del11p), plasma membrane protrusions, cytoskeleton systems [40,41], adhesion molecule systems such as CXCL12/CXCR4 (chemokine ligand 12/chemokine receptor 4) [42], junctional adhesion molecules in osteotropic tumors [43,44,45], focal adhesion kinases [46,47,48], and vascular and immune-microenvironment interactions [49,50,51,52].

**Figure 1:**
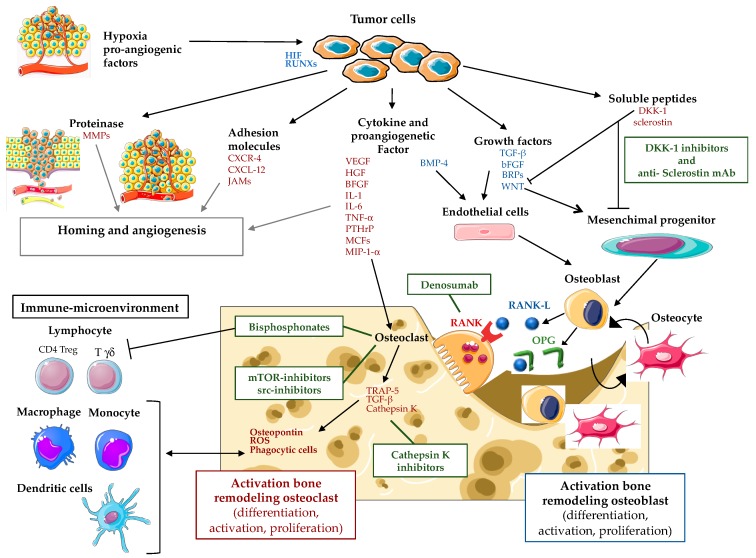
Bone metastasis physiopathology: implication of cancer cell-microenvironment interactions and therapeutic targets. HIF, hypoxia-inducible factors; RUNXs, Runt-related transcription factors; MMPs, matrix metallopeptidases; CXCR-4, chemokine receptor 4; CXCL-12, chemokine ligand 12; JAMs, junctional adhesion molecules; VEGF, vascular endothelial growth factor; HGF, hepatocyte growth factor; BFGF, basic fibroblast growth factor; IL, interleukins; PTHrP, parathyroid hormone-related protein; MCFs, macrophage chemotactic factors; MIP-1, macrophage inflammatory proteins 1-alpha; BMP-4, bone morphogenetic protein 4; TGF-β, transforming growth factor beta; BFGF, basic fibroblast growth factor; BRPs, bone resorptive proteins; WNT, wingless-related integration site; DKK-1, Dickkopf WNT signaling pathway inhibitor 1; RANK, receptor activator of nuclear factor-kappaB; RANKL, receptor activator of nuclear factor-kappaB ligand; OPG, osteoprotegerin; TRAP-5, tartrate resistant acid phosphatase 5; mTOR, mammalian target of rapamycin.

Based on the knowledge acquired, mesenchymal cell numbers, their immune modulatory effects, and their interactions with matrix molecules play a pivotal role in establishing a critical dependency between cancer cells and the tumor niche. Breast and prostate cancer cells home to the bone marrow, where they presumably hijack the hematopoietic stem cell niche [53] and develop metastatic lesions. These lesions are well known tumors that educate the metastatic environment to guide the dissemination process [35]. Nonetheless, the premetastatic niche remains elusive in the context of tumors of unknown origin. Mesenchymal stromal cells (MSCs) are permissive in cancer cell homing and the growth of bone metastases when pharmacologically decreased in number, enhancing cancer cell homing to the bone marrow in mice [54]. In the complex relationship between the tumor cells, the organ specific vasculature [55], and their microenvironment, an insufficient oxygen supply (hypoxia) is a prominent feature in various pathological processes, including tumor development and metastasis [56,57]. The central mediators during hypoxia are hypoxia inducible factors (HIF), whereas their downstream effects are closely regulated by oxygen-dependent HIF prolyl hydroxylases (PHDs). PHD2 plays a central role during the different stages of tumor development, whilst this oxygen sensor is also essential during bone mineralization and normalization of the endothelial barrier in the bone (marrow) after stress [58]. Moreover, the perivascular niche directly influences the equilibrium between dormant tumor cells, their retention, and cancer reactivation [35]. In more detail, the cancer dissemination to the bone shares common steps comprising various stages. They include cell proliferation at the site of the primitive outbreak, penetration in the blood and lymphatic vessels, anchoring of an anatomical cell away from the original outbreak to the basal membrane, parenchyma infiltration of the new anatomical site, and proliferation. The process is actively supported by neoangiogenesis via cytokines such as vascular endothelial growth factor (VEGF), basic fibroblast growth factor (BFGF), and transforming growth factor-alpha (TGF-α). The neo-vessels are structurally fragile and different from normal ones in that the basal membrane and extracellular matrix are easily destroyed by proteases produced by cancer cells, such as metalloproteases (MMPs), cathepsin D, and plasminogen activator, resulting in extravascular passage of cancer cells. Cancer cells also modify the adhesive capabilities and their mobility through their own factors, hepatocyte growth factor/stromal factor (HGF/SF) and insulin growth factor II (IGF-II), or by matrix proteins such as vitronectin, fibronectin, laminin, and type IV collagen, as well as host-secreted factors such as insulin growth I (IGF-I), interleukin 6 (IL-6) and histamine. Finally, the location of bone-level cancer cells is determined by the response to chemotactic stimuli by the cellular components activated by type I collagen, osteocalcin, or cytokines such as transforming growth factor-beta (TGF-β) and platelet-derived growth factor (PDGF). The high affinity shown by cancer cells for bone is due both to high vascularization and to the fact that the bone microenvironment frees up factors that promote their survival and proliferation. Once cancer cells reach the bone marrow level, they cross the wall of sinusoids, invade the matrix, and, once they reach the endosteal surface, stimulate osteoclastogenic activity and proliferate with the formation of metastases with prevailing osteolytic or osteoblastic development. These extremes cover the spectrum of neoplastic bone remodeling (Figure 1) [35,59].

The knowledge of the underlying mechanism of bone dissemination already prompted an extensive clinical investigation. Nonetheless, skeletal metastases remain a poor prognostic event with huge morbidity and mortality impacts [7,8].

## 3. Clinical Management for Skeletal Metastasis of Unknown Primary

### 3.1. Diagnostic Work-Up of SMUP

**Figure 2:**
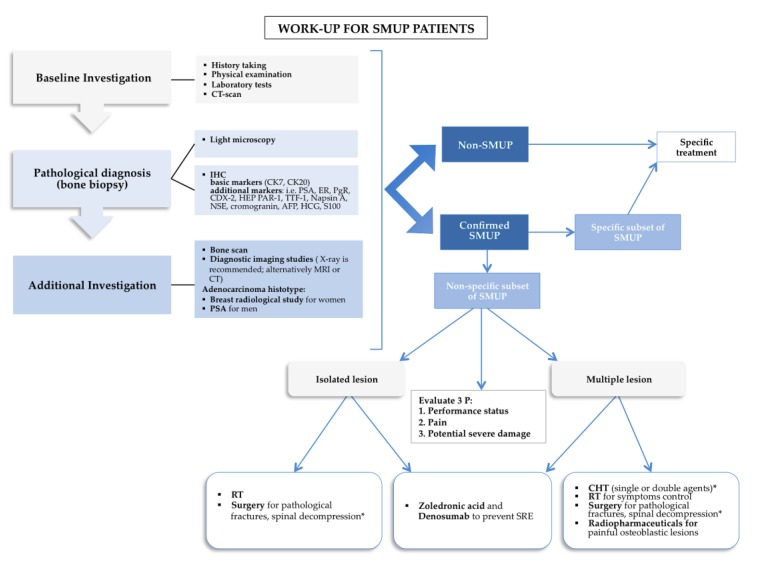
Stepwise-clinical management of the patient with skeletal-metastasis of unknown primary (SMUP) suitable to individualized approaches. CT, computer-tomography; CK, cytokeratin; PSA, prostate-specific antigen; ER, estrogen receptor; PgR, progetseron receptor; CDX-2, caudal type homeobox 2; HEP PAR-1, hepatocyte specific antigen; TTF-1, thyroid transcription factor 1; NSE, neuron-specific enolase; AFP, α-fetoprotein; HCG, human chorionic gonadotropin; RT, radiotherapy; CHT, chemotherapy; IHC, immunohistochemistry. *: depending on performance status.

Skeletal metastases represent a clinical challenge regarding the diagnostic work-up for patients suffering from CUP. Clinical judgment and approaches borrowed from CUP represent a reasonable pragmatic alternative and a valid paradigm to design statistically-powered clinical studies. Indeed, minimal basic work-up for SMUP overlaps with overall CUP when it includes medical history, physical examination, basal blood and biochemical analysis (including bone metabolism), and computer-tomography (CT) scans of the thorax, abdomen, and pelvis [60,61,62]. Integrative investigation must be selected based on clinical and radiological indications, such as endoscopy and serum assessment of prostate-specific antigen (PSA), α-fetoprotein (AFP), β-human chorionic gonadotropin (β-HCG), and chromogranin to exclude “treatable” or susceptible hormone therapy and can drive site-specific treatment [29]. However, the tumor biopsy remains a pivotal point in the SMUP diagnostic process, providing tissue suitable for light-microscopic and immunohistochemical examination and molecular characterization [60,61]. Further consequential, detailed, practical, and pathological primary and specific markers are summarized in Figure 2. Additional molecular investigations, such as gene-expression profiling (GEP) assays, hold the promise to characterize more deeply the underlying malignancies, guide a tailored therapy, and identify the tissue of origin in patients with occult primary cancers [20,63,64,65]. Immunohistochemistry (IHC) and GEP offer a similar range of accuracy in tumor classification (approximately 75%) [66]. Nonetheless, the quality of evidence available is not strong enough to allow stringent recommendations and selected classifier assays. Approaching the differential diagnosis of suspected adenocarcinoma, PSA and mammografy are two effective screening procedures for men and women, respectively [67]. Breast magnetic resonance imaging (MRI) and ultrasound can efficiently complete non-diagnostic screening procedures. Among additional investigations (Figure 2) whole-body radionuclide bone scans are deemed as sensitive techniques, despite being non-specific, providing information on osteoblastic lesions and bone vascular density, with a selective signal dependent on skeletal osteoblastic remodeling, either neoplastic, inflammatory, or post-injury [68]. Conversely, lytic bone lesions are better characterized by conventional radiology (X-ray), CT, and MRI than by bone scan, due to the lower metabolic extent within the skeletal compartment compared to osteoblastic tumors [68,69]. X-ray, CT, and MRI bone scans can also be used in case of painful lesions or bone scan positivity that requires further targeted investigation, holding the potential to clarify the etiology of weight-bearing imaging areas [66,70]. It has been shown that ^18^F-fluorodeoxyglucose positron emission tomography (^18^F-FDG-PET)-scan and single-photon-emission-tomography (SPECT) can both significantly enhance the diagnostic accuracy [71], supporting the primary sites investigation in 37% of cases [72].

Literature data show that the minimal basic work-up allows for the identification of the primary tumor in about half of the cases of SMUP. In particular, Takagi et al. reported that CT scans discovered 30% of SMUP. Bone biopsy and origin examination identified another 19% and 14% of primary sites, respectively. Bone marrow puncture and PET added only 1% of the diagnoses each [9]. Despite the improvement of diagnostic methods, the primary site is not always identified.

### 3.2. Therapeutic Approach of SMUP

An unfulfilled medical need of real-life clinical practice in identifying the primary cancer, deeply impacts the choice of therapy. Therefore, in order to face this challenging task, gene-expression signatures investigation along with pathological characterization have been employed with the goal of gaining an enhanced clinical survival [20,60,73]. Along these lines, a site-specific therapy integrated with a GEP-guided treatment was shown to improve the clinical outcome [61,74].

From this standpoint there is exclusion of both a non-CUP and a specific subset of CUP-deserving site-oriented therapies. Next, risk-driven therapy represents the cornerstone of the clinical-judgment directed-treatment approach. Therefore, CUP patient stratification into prognostically favorable and unfavorable groups allows differential clinical management of poorly differentiated cancer of the midline, papillary peritoneal cavity cancer of adenoma subtypes in females, adenocarcinoma with isolated involvement of axillary lymph nodes in women, and cancer of squamous type involving neck nodes [75,76,77]. Nonetheless, more than 80% of CUP are deemed to be prognostically unfavorable, showing uncertain chemotherapy response [78]. In the last few years, prognostic scores able to homogeneously categorize CUP patients have been explored, including performance status (PS) evaluation and serum biomarkers sharing common features with known primary bone metastatic cancers (i.e., lactate dehydrogenase and albumin) [78,79,80,81,82]. Bone metastasis, per se, represents a prognostic factor for survival of CUP [7,8]. There are no parameters to detect significant differences in terms of clinical outcome in SMUP with low PS and biomarkers (with about 1 year of life expectancy) compared to poor prognosis features, such as PS >2 and elevated biomarkers level. The latter group of subjects could profit the most from a palliative approach, given an mOS of 4 months [2]. An additional independent prognostic factor is the number of bone lesions that can also imply a differential therapy management constituted by integrated modalities of radiotherapy and surgery when either a single bone lesion or painful clinical scenario are present [5]. A surgical approach should be reserved for patients with good PS, when acute complications, such as pathological fracture, require spinal decompression [5].

In patients with extensive metastatic disease, metabolic radiotherapy, given its systemic distribution, represents a valid therapeutic option. Bone-seeking radiopharmaceuticals have been employed as active osteotropic approaches [83], representing calcium mimetics in the bone compartement, which emit alpha, beta, and gamma particles. Strontium-89 chloride, Samarium-153-ethylenediamine tetramethylene phosphonic acid (EDTMP), Rhenium-186-hydroxyethylidine diphosphonic acid (HEDP), and Radium-233 dichloride were indicated for the treatment of painful lesions due to osteoblastic or mixed bone metastases, mainly from breast and prostate tumors and other cancers with osteoblastic skeletal spreading, detected by whole-body bone scans performed ahead of treatment [83]. These compounds mainly target osteoblastic skeletal lesions, however, they are not effective on lytic bone lesions, increasing the fracture incidence [84].

Remarkably, risk fracture implies an additional fundamental criterion to guide a multistep integrated management. Conversely, cytoreduction along with PS stratification can guarantee a more effective treatment in bone diffuse involvement (Figure 2).

Given the poor effectiveness of chemotherapeutic agents and the results derived from metanalysis, cytoreduction should be undertaken only in symptomatic disseminated disease, when PS allows for the aforementioned approaches. In all cases, the aim of the treatment should be represented by an improvement in the quality of life and symptom control with the maximal minimization of the toxicity profile. In the absence of standardized high-quality evidence, the regimen of choice should be based on the histopathological data. More specifically, platinum-, taxan-, gemcitabine- and irinotecan-based regimens constitute the backbone of combination therapies with proven efficacy [85,86,87].

Consistent and high quality clinical evidence constitutes an unmet clinical need, due to the lack of prospective controlled, randomized trials designed to gain pragmatic insights from both clinical studies and real-life practice.

### 3.3. Bone Disease Modifying Agent and Bio-Marker Reciprocal Interconnections

#### 3.3.1. Bisphosphonates and Bone Disease Modifying Agents

A milestone in the clinical management of skeletal related events (SRE) in cancer, including SMUP, were the discovery of bisphosphonates and SRE-directed therapies. Bisphosphonates are effective in increasing survival and reducing skeletal complications, including hypercalcemia, delaying the time of appearance of skeletal complications, and reducing bone pain in patients with metastases, particularly intravenous ones [88,89,90,91,92].

Denosumab is a viable subcutaneous alternative and a preferred option as it has shown moderate greater efficacy than Zoledronic acid in reducing skeletal events (excluding hypercalcemia) [93,94].

Several molecules with direct or indirect effects on the bone metastases progression of solid tumors are currently being investigated in several clinical trials. Some of these molecules are able to act directly on the bone resorption process, targeting specific bone cells such as osteoclasts, osteoblasts, osteocytes, or molecular pathways that regulate the function of these cells. This group includes denosumab inhibitors of the endothelin 1 receptor (also expressed by osteoblasts), the inhibitors of cathepsin K (produced by osteoclasts, but also by metastatic tumor cells to the bone), drugs that interfere with the wnt/dkk1 pathway (which, among other things, regulates the function of osteoblasts and immune-cells), and src inhibitors (non-receptor tyrosine kinases downstream of the rank receptor, which help regulate the resorbing function of osteoclasts), but to date, the main point of these drugs that has been considered is their function in stopping the destruction of the bone matrix by the tumor cells [59,95,96,97,98,99]. The optimal treatment duration has not been established. Clinical trials with zoledronate and ibandronate have shown a benefit for a treatment period of at least 2 years [100,101,102,103]. Moreover, irrespective from the appearance of a skeletal event, the continuation of Zoledronic acid therapy has been demonstrated to hold a statistically significant reduction in the occurrence of subsequent events [104].

Prolonged treatment with Zoledronic ccid of more than two years, besides being associated with a low SRE rate, is characterized by a good safety profile [105].

J. P. Winters et al. presented a retrospective analysis showing that the risk of SRE was greater in the first two years of treatment in a cohort of 92 patients with bone metastases from solid tumors and with Multiple Myeloma treated with Pamidronate or Zoledronate for over two years (average duration of 36 months) [106]. The toxicity profile was acceptable for both drugs, regardless of the duration of treatment.

The therapy duration is extremely heterogeneous, varying from 12 weeks (in the first phase of study of denosumab) [107] to 96 weeks (for bisphosphonates) [100,108,109,110] up to about 34 months in phase III studies on denosumab in breast and prostate cancer [111,112]. These studies have not provided compelling evidences regarding the optimal duration of treatment without elucidating the comparison between continuous or interrupted therapy.

Patients suffering from breast or prostate cancer that are enrolled in phase III studies of denosumab have been asked to participate in a subsequent study on long-term treatment [113]. Patients chose whether to continue with a further two years of treatment with denosumab or proceed with two years of follow up. A total of 948 patients accepted the continuation of therapy, reaching a maximum duration of treatment of about five years in patients with breast cancer and about 5.6 years in patients with prostate cancer. The results of the study showed a good tolerability in the treatment with denosumab both after prolonged exposure and after switching to denosumab following previous Zoledronic acid therapy [113].

In consideration of the aforementioned evidence, in the absence of specific data and statistically powered clinical studies able to identify an optimal treatment period, the currently recommended duration for bone target therapy is at least two years, suspending treatment in case of deterioration of PS. Continuation of treatment beyond the two-year limit is however recommended (especially in the case of denosumab therapy), taking into account the risks of developing skeletal events, tolerability, and general clinical conditions of the patient [93,114,115,116,117].

#### 3.3.2. The Role of Bone Turnover Markers in Diagnosis and Therapy Response with Inhibitors of Bone Resorption Evaluation

As markers of bone turnover, it is advisable to dose “bone turnover markers”, defined as degradation products of type I collagen specific to bone tissue, that circulate after osteoclastic digestion or pro-collagen type 1 cleavage, and represent the expression of the neoformative synthesis activity of osteoblasts. Alkaline phosphatase (bone isoenzyme) is an enzyme that is an expression of osteoblastic activity, similar to osteocalcin. Parathyroid hormone (PTH), calcemia, and vitamin D are not strictly markers of osteoblastic and osteoclastic cellular activity [118,119]. The recent important role reserved for osteocytes has led to the emergence of new potential markers, such as Dickkopf WNT signaling pathway inhibitor 1 (DKK1), sclerostin, cathepsin K, and tartrate-resistant acid phosphatase 5 (TRAP-5), which are very promising, but still reserved for research activities. The bone turnover markers currently considered the gold standard are P1NP (pro-type collagen) for neoformation activity and CTX (terminal p-type peptide of type 1 collagen) for osteoclastic resorbing activity [118,119]. Their clinical use in metabolic diseases of the skeleton, primarily postmenopausal osteoporosis, is still limited and is not recommended by the guidelines [120].

A potential use of bone turnover markers is to be able to drive the diagnostic investigation of SMUP. Numerous studies have shown, in a cross-sectional manner, the association between high levels of CTX and N-terminal telopeptide (NTX) and the presence of bone metastases [68,119,121]. However, a specific marker with sufficient discriminated power was not highlighted. It should be considered that patients with bone metastases generally have a very high bone turnover for many reasons (D hypovitaminosis, age, hormonal therapy, etc.). Therefore, the specificity and accuracy of the biomarkers can be affected by non-neoplastic sources, which differ from the metastatic site. Even in combination with diagnostic methods their sensitivity is not high, and their clinical use is not indicated [68,121].

The prediction of skeletal complications of bone metastases (SRE or SSE) is a potential clinical use of bone turnover markers, since the level of markers can be correlated to the extent and activity of the metastases themselves. From meta-analysis and post-hoc analysis of randomized clinical trials with zoledronic acid, some markers such as NTX and alkaline phosphatase (ALP) bone have been shown to be able to predict not only SRE but also the progression of metastatic bone disease and survival. This resulted both with the basal values (in the absence of therapy) and based on the response (after three months) to the treatment with zoledronic and denosumab [122]. This data is confirmed several times in the literature. For example, the levels of bone ALP and also of P1NP correlate with the progression of metastatic disease at the bone level and with survival in urologic cancers, such as prostate cancer [123]. To confirm this, ALP and NTX were associated with survival in patients with bone metastases due to prostate cancer in the hormone-resistant phase [124]. Similar data have also been reported in other neoplastic histotypes and in tumors of unknown origin [125,126].

Another potential use of bone turnover markers could be to monitor the effectiveness of zoledronic therapy. The reduction of bone turnover markers during therapy with resorption inhibitors can be used as a surrogate to evaluate efficacy on pain, and on the risk of SRE. A failure to normalize bone turnover during therapy with bone resorption inhibitors could indicate an optimal sub-effect of the same therapy. Fizazi et.al. studied women with breast cancer who did not normalize bone turnover with zoledronic acid; these women were randomized to receive denosumab or continue with zoledronic acid. In the transition to denosumab, a rapid and complete normalization of the NTX values was obtained with an advantage in terms of reduction of SRE [127]. Despite there being few clinical trials in patients with unknown origin, in the patients with SMUP, the baseline value of the bone metabolism markers and their decrease during treatment might be used with caution as a predictor of SRE, bone progression, and survival [128,129]. 

## 4. Conclusions

SMUP represents a diagnostic and therapeutic clinical need that remains unfulfilled; there are few evidence-based indications available about the tight underlying relationship between the challenge of diagnostic work-up and the treatment choice. In addition to the surgical approach and radiotherapy, the systemic option available for patients diagnosed with SMUP is represented by bone modifying agents and chemotherapy. Nonetheless, currently it is mandatory to encourage patient-enrollment in clinical trials involving the characterization of the genetic and molecular profiles of SMUPs and to integrate standard chemotherapy associated with SRE therapy with molecular target agents. 

## Figures and Tables

**Table 1:** Bone metastases of unknown primary data overview and corresponding identified primaries and survival implications.

Authors	SMUP at Diagnosis	Identified Primary Cancer	Number and Site of Primary Cancer Identified	Confirmed SMUP	mOS Confirmed SMUP (*Months*)
Simon and Karluk [10]	*n* = 12	*n* = 6 (50%)	Kidney (3), lung (2), others (1)	*n* = 6 (50%)	NA
Simon and Bartucci [11]	*n* = 46	*n* = 20 (44%)	Lung (7), kidney (6), breast = prostate (2), ovarian/thyroid/liver (1)	*n* = 26 (56%)	NA
Nottebaert et al. [12]	*n* = 51	*n* = 33 (65%)	Lung (17), others (16)	*n* = 18 (35%)	11.1
Shih et al. [13]	*n* = 52	*n* = 28 (54%)	Lung (9), liver (8), kidney (5), prostate (3), thyroid (2), rectum (1)	*n* = 24 (46%)	11
Rougraff et al. [14]	*n* = 40	*n* = 34 (85%)	Lung (23), kidney (4), breast/colon/liver/ bladder (1), others (3)	*n* = 6 (15%)	NA
Jacobsen et al. [15]	*n* = 29	*n* = 24 (83%) (2 patients postmortem)	Lung (11), prostate (3), breast/lymphomas (2), kidney/ovary/pancreas/stomach/small intestine carcinoid/retroperitoneal rhabdomyosarcoma (1)	*n* = 5 (17%)	12
Katagiri et al. [16]	*n* = 64	*n* = 59 (92%)	Lung (23), prostate (11), breast/liver (5), others (15)	*n* = 5 (8%)	5
Vandecandelaere et al. [17]	*n* = 129	*n* = 84 (65%)	Lung (36), prostate (17), kidney (15), breast (9), stomach (2), bladder/colon/testis/pancreas/liver (1)	*n* = 45 (35%)	6
Destombe et al. [6]	*n* = 152	*n* = 94 (88%)	Lung (37), prostate (26), breast (20), urinary system (11)	*n* = 13 (12%)	NA
Iizuka et al. [18]	*n* = 27	*n* = 26 (96%)	Myeloma (7), lymphoma (3), lung (6), prostate (4), kidney/thyroid/liver/pancreas/stomach/esophagus (1)	*n* = 1 (4%)	NA
Hemminki et al. [8]	*n* = 501	*n* = 256 (60%)	Lung (128), urinary (29), prostate (16), breast (14), colon (12), pancreas/gastrointestinal (10), liver (9), biliary system (4), stomach (3), mediastinum (2), others (19)	*n* = 203 (40%)	3
Takagi et al. [9]	*n* = 286	*n* = 254 (89%)	Lung (72), myeloma (41), prostate (26), lymphoma (23), kidney (18), liver (12), breast (12), gastric (10), pancreatic (10), thyroid (9), bile duct/colon (6), esophageal (3), others (6)	*n* = 32 (11%)	11

SMUP, skeletal metastasis of unknown primary; mOS, median overall-survival.
